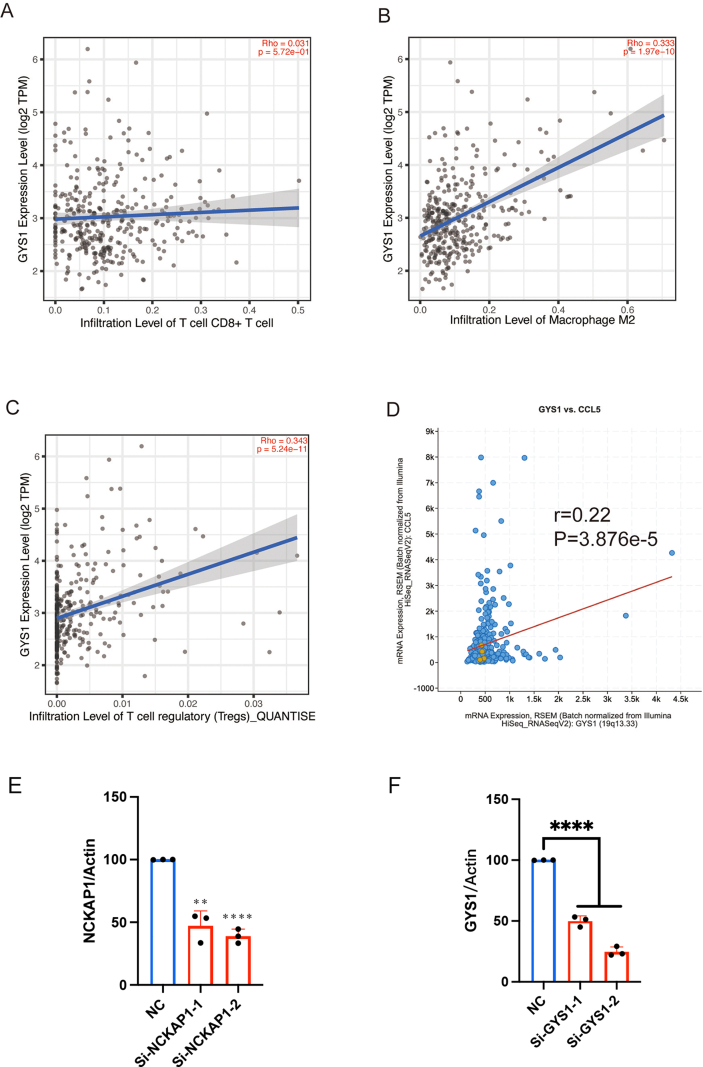# Identification and validation of a disulfidptosis-related genes prognostic signature in lung adenocarcinoma

**DOI:** 10.1016/j.heliyon.2023.e23502

**Published:** 2023-12-19

**Authors:** Yanpeng Zhang, Jingyang Sun, Meng Li, Liren Hou, Zhiyu Wang, Huanhuan Dong, Wenjun Xu, Rongxuan Jiang, Yuhan Geng, Chungen Guan, Zijiang Zhu, Hongyi Wang, Qiuyu Gong, Guangjian Zhang

**Affiliations:** a Department of Thoracic Surgery, The First Affiliated Hospital of Xi'an Jiaotong University, Xi'an, China; b The First Clinical Medical College of Gansu University of Chinese Medicine (Gansu Provincial Hospital), Lanzhou, China; c Department of Respiratory and Critical Care Medicine, The First Affiliated Hospital of Xi'an Jiao Tong University, Xi'an, China; d Department of Thoracic Surgery, Gansu Central Hospital, Lanzhou, China

**Keywords:** Prognostic signature, Lung adenocarcinoma, Disulfidptosis, LASSO

## Abstract

Disulfidptosis, a newly revealed form of cell death, regulated by numerous genes that has been recently identified. The exact role of disulfidptosis in lung adenocarcinoma (LUAD) still uncertain. Objective of this study was to explore potential prognostic markers among disulfidptosis genes in LUAD. By combining transcriptomic information from Gene Expression Omnibus databases and The Cancer Genome Atlas, we identified differentially expressed and prognostic disulfidptosis genes. By conducting least absolute shrinkage and selection operator with multivariate Cox regression, four disulfidptosis genes were selected to create the prognostic signature. The implementation of the signature separated the training and validation cohorts into groups with high- and low-risk. Subsequently, the model was verified by conducting an independent analysis of receiver operating characteristic (ROC) curves. Further comparisons were made between the two risk-divided groups with regards the tumor microenvironment, immune cell infiltration, immunotherapy response, and drug sensitivity. The signature was constructed using four disulfidptosis-related genes: SLC7A11, SLC3A2, NCKAP1, and GYS1. According to ROC curves, the signature was effective for predicting LUAD prognosis. In addition, the prognostic signature correlated with sensitivity to chemotherapeutic agents and the efficacy of immunotherapy in LUAD. Finally, through external validation, we showed that NCKAP1 are correlated with tumor migration, proliferation, and invasion of LUAD cells. GYS1 affects immune cell, especially M2 macrophage infiltration in the tumor microenvironment. The disulfidptosis four-gene model can reliably predict the prognosis of patients diagnosed with LUAD, thereby providing valuable information for clinical applications and immunotherapy.

## 1 Introduction

Lung cancer is the leading cause of cancer-related death worldwide, killing 1.3 million people annually metastases form in more than half of patients with lung cancer. It is important to remember that just 6 % of people with metastatic lung cancer survive for five years [1]. Compared to other lung cancer subtypes, lung adenocarcinoma (LUAD) is the predominant histological subtype [2]. Chemotherapy, radiotherapy, and drug therapy are all current treatments for LUAD [3]. However, a considerable proportion of patients with LUAD are not identified until the advanced phase, thereby losing the prospect of surgical intervention. In some cases, metastasis may have already occurred, further limiting treatment options [4]. Since there are more choices for the treatment of early stages of LUAD, early and clear diagnosis of LUAD and its timely treatment will increase the survival rate of patients with LUAD. Although early computed tomography screening has potential advantages in detecting and managing LUAD, the efficacy and outlook of conventional therapeutics remain unsatisfactory, primarily because of the peculiar invasiveness and pharmaceutical resistance of this disease [5,6].

Modern research has emphasised the several forms of regulated cell death (RCD), such as apoptosis, ferroptosis, pyroptosis, and cytoproptosis. Each RCD type exhibits unique morphological, biochemical, and functional traits, the boundaries between these RCD types are not always clear, and they may intersect and overlap under certain circumstances [7]. RCD can be roughly divided into cell “suicide” and “sabotage” programs [8]. A newly discovered type of RCD, known as disulfide stress-induced cell death, has been identified. It is marked by the disulfide bonds’ abnormal buildup in intracellular molecules and proteins, leading to the coining of the term “disulfidptosis” to describe this process. Given that this mechanism induces cell death through disulfide stress, it is likely a cellular “disruption” program [9]. Although disulfidptosis represents a novel form of RCD, the precise mechanisms by which disulfide stress triggers cell death in tumors remain unclear, and the clinical relevance of disulfidptosis in LUAD requires further investigation. Therefore, additional research is required to address this gap.

The main objective of this research was to utilize bioinformatics techniques to analyze the transcriptome information and prognostic implications of disulfidptosis genes in LUAD. Our findings provide a sound basis for appraising the prognostic importance of disulfidptosis-associated genes in LUAD and for devising a signature for targeting disulfidptosis to prevent and manage this disease.

## 2 Materials and methods

### 2.1 Data collection and pretreatment

The LUAD initiative from the Cancer Genome Atlas (TCGA) repository offered preprocessed RNA-sequencing (RNA-seq) information, encompassing 542 tumor and 58 normal samples. The clinical Data from the TCGA-LUAD project was acquired for a total of 522 patients. The training cohort consisted of the patients who were included after excluding thirteen patients because they lack of overall survival (OS) data, five patients without RNA-seq data.

In order to test the prognostic signature and to avoid data bias, the datasets GSE30219 [10] and GSE31210 [11] the Gene Expression Omnibus (GEO) were utilized. To make the merged data available for analysis and ensure that the merged data had a certain level of quality and comparability, both datasets in the validation cohort were obtained from the same platform, GPL570. The normalized series matrices for the two RNA microarrays were retrieved from GEO: GSE30219 consisted of 85 LUAD tumor samples and GSE31210 consisted of 226 LUAD tumor samples. Data merging and removal of batch effects were relied on the dataset utilizing the “inSilicoMerging” R package and the method of Johnson WE et al. [12]. RNA-seq information from TCGA and the series matrices from GSE30219 and GSE31210 were processed using the “log 2 (normalized gene expression + 1)” method. Samples containing “NA” were removed and missing values were completed using the “impute” R package. All data used were publicly accessible, and the policies and guidelines for publications set forth by TCGA and GEO databases were strictly adhered to. In total, ten disulfidptosis genes were compiled from previous studies [9] and shown in Supplementary Table 1.

### 2.2 Signature construction

Mann–Whitney test was performed by using “Wilcox.test”, “limma” R packages, to extract disulfidptosis-related differentially expressed genes (DEGs). Based on OS in the training cohort, we performed univariate Cox regression to evaluate the predictive value of the disulfidptosis-related genes. The prognostic disulfidptosis-related genes and disulfidptosis-related DEGs were then intersected, and the overlapping genes were considered prognostic disulfidptosis-related DEGs. The training datasets for the prognostic signature were derived from tumor tissues in TCGA-train cohort. The least absolute shrinkage and selection operator (LASSO) regression method was used to identify potential genes. This process determined an optimal penalty parameter λ for each gene towards establishing the coefficient for risk score calculation. Finally, a linear combination of variables was used to derive a formula. The model was constructed by using this formula [13]:

RiskScore=N∑i=1(exp*coef)

where “N” stands for gene number, “exp” represents the gene expression, “coef” reflects corresponding coefficient of the gene. Using this model, patients with TCGA-LUAD were categorized into high-/low-risk groups. For comparison, Kaplan-Meier survival analysis was performed. In order to assess the sensitivity of the signature, the receiver operating characteristic (ROC) curve was constructed using the “timeROC” R package (version 4.21). Principal component analysis (PCA) and t-distributed stochastic neighbor embedding (*t*-SNE) were employed to uncover intrinsic features within these two cohorts using the R software packages “stats” and “Rtsne,” respectively. Univariate and multivariate Cox regression, independently analyze the risk score, stage, sex, age, and risk-score in two cohorts. By utilizing the identical approach as the one utilized in the LASSO technique, we calculated a risk assessment for every individual and subsequently categorized patients from the GEO database into groups of high or low risk.

### 2.3 Nomogram construction

The survival rates of LUAD patients were predicted using risk scores and clinical information using a nomogram. Calibration curves could assess the concordance between predicted and observed OS probabilities. To validate the precision of the signature, Concordance Index (*C*-index) curves were employed.

### 2.4 Functional enrichment analysis

The R software and its affiliated packages “org.hs.eg.db”, “clusterprofiler”, and “ggplot 2” were utilized to perform Gene Ontology (GO) and Kyoto Encyclopedia of Genes and Genomes (KEGG) pathway analyses. Based on *Homo sapiens*, and screening criteria of p. adjust <0.1 and p-value <0.2 were used to identify the primary enriched functions and pathways.

### 2.5 Immune microenvironment patterns between risk groups

CIBERSORT, utilizes transcriptome data to approximate the quantity of 22 unique immune cells in the expression data of a bulk tumor sample [14]. This estimation was performed using linear support vector regression. To evaluate immune-related functions, we used ssGSEA analysis with the aid of the “GSVA” R package. The correlation analysis of GYS1 involved in the signature with CD8^+^ T cell, Treg, and M2 macrophage infiltration was conducted using TIMER2.0 [15] (http://timer.cistrome.org/). cBioportal [16] (https://www. cbioportal. org/). The correlation between GYS1 expression and the chemokines colony-stimulating factor-1 (CSF1) and *c*-c chemokine ligand 5 (CCL5) was analyzed using the organization's tool.

### 2.6 Evaluation of drug sensitivity and efficacy of immunotherapy

We used the “oncoPredict” R package to calculate the 50 % maximum inhibitory concentration (IC50) of diverse sample sets using ridge regression to forecast their response to chemotherapy [17]. To compare the IC50 values of distinct groups, we employed the Wilcoxon signed-rank test. TIDE (http://tide.dfci.harvard.edu/) employs transcriptome information profiles to assess the tumor microenvironment (TME) [18] and has proven useful for forecasting immune checkpoint blockade (ICB) therapy [19,20].

### 2.7 Cell culture

The cells were placed in an environment with 95 % humidity, 5 % CO2, and incubated at a temperature of 37 °C. The H1650, A549, H1299, H460 and PC9 cell lines of human lung cancer, as well as BEAS-2B cells of normal human alveolar epithelial, were procured from the American Type Culture Collection (ATCC, Manassas, U.S.A.). The H460, H1650, H1299, and PC9 were cultured in RPMI-1640 medium with 10 % FBS, while A549 and BEAS-2B were cultured in DMEM with 10 % FBS. Cells proliferation, migration, invasion, and clone formation assays were conducted on H460 human LUAD cells.

### 2.8 siRNA construction and infection

Tsingke Bio Technology Co., Ltd. (Beijing, China) designed and manufactured the siRNA sequences. Lipofectamine 3000 (Invitrogen) was used to introduce siRNA into the culture medium of LUAD cells. The targeted NCKAP1 knockdown sequences were as follows: *Si*-NCKAP1-1, (5′-CAUCCUAUCUUAUCGACAA-3′) and *Si*-NCKAP1-2, (5′-CAGACGACUUUAUAGAUAA-3′); GYS1 knockdown sequences were *Si*-GYS1-1, (5′-CUCGAAUCCAGGAGUUUGUTT -3′) and *Si*-GYS1-2, (5′-GGACACUGGAUUCCAUGAATT-3′).

### 2.9 Cell counting kit-8 assay

A 96-well plate was utilized in the CCK-8 trial. Each well was filled with 15 μL of CCK-8 reagent. Afterwards, the plate was incubated for 1 h to facilitate the interaction between the cells and the CCK-8 reagent. After the incubation period, each well's absorbance at a wavelength of 450 nm was measured using a microplate reader. The absorbance measurement at a wavelength of 450 nm is directly correlated with the quantity of living cells, indicating their level of metabolic functioning.

### 2.10 Immunohistochemical (IHC) staining

As previously described [21], the paraffin-embedded microarray tumor tissues using IHC primary antibodies, including SLC7A11 (Proteintech 26864-1-AP), SLC3A2 (Proteintech 15193-1-AP), NCKAP1 (Proteintech, 12140-1-AP), and GYS1 (Proteintech 10566-1-AP) antibodies, along with their corresponding non-cancerous tissues. The lung cancer tissue microarray was acquired from Shanghai Outdo Biotech Co., Ltd. (Shanghai, China; HLugA060PG02). After baking at 60 °C for 30 min, paraffin-embedded LUAD tissues were dewaxed. Treatment with 3 % hydrogen peroxide for 15 min resulted in the inhibition of the inherent peroxidase activity. After undergoing antigen restoration, the microwave tissues were sealed with fetal bovine serum for a duration of 30 min and subsequently incubated at a temperature of 4 °C overnight. Following the period of incubation, the tissues underwent treatment with secondary antibodies conjugated with horseradish peroxidase, maintaining a temperature of 37 °C for a duration of 60 min. Following the washing process, the sections were observed utilizing the DAB detection kit. The tissues were evaluated by two pathologists who worked independently and were not aware of the clinical parameters. Any differences in their opinions were resolved through consensus with the help of a third observer.

### 2.11 Western blotting

As previously described [22], prepared cells were added with pre-cooled RIPA buffer (Beyotime, Shanghai, China) and supplemented with protease and phosphatase inhibitors. The resulting samples were denatured by heating to 100 °C for 15 min. The BCA™ kit (Thermo Fisher Scientific, USA) was used to measure protein concentration. The same quantity of protein was increased in size and placed onto a gel made of 12 % sodium dodecyl sulfate-polyacrylamide, then moved to a PVDF membrane. The membrane was placed in a solution of tris-buffered saline with 5 % (w/v) milk in tween and incubated for 1.5 h. The primary antibody was included and left to incubate at a temperature of 4 °C for the entire night. Afterwards, the membrane was subjected to the secondary antibody for an additional hour. Enhanced chemiluminescence was utilized to detect the immunoblots ultimately. Primary antibodies were GYS1 (Proteintech, 10566-1-AP) and NCKAP1 (Proteintech, 12140-1-AP).

### 2.12 Transwell assay detects cell invasion ability

In the Transwell assay, cells were seeded in the upper chamber of an 8 μm pore size Transwell insert coated with matrix adhesive. The bottom chamber was filled with a medium for growth. Following a 24-h incubation period, the stationary cells located on the top side of the membrane were eliminated. Crystal violet was used to fix and stain the migrated cells on the lower surface.

### 2.13 Clone formation assay

In each group, 1000 cells were placed into 12-well plates with 3 replicate wells. The plates were then placed in a 37 °C incubator with 5 % CO2 for cultivation. The liquid was changed per 3 days. After approximately two weeks of cultivation, clones were formed when cell colonies became visible to the naked eye. The cells underwent treatment using 4 % paraformaldehyde for a period of 30 min, then proceeded to be stained with 0.1 % crystal violet for a duration of 20 min. Afterwards, the cells underwent washing and drying with PBS buffer to remove any remaining dye and non-specific compounds. Finally, the clones were imaged and counted.

### 2.14 Wound healing assay

To form a full layer of cells, cells were arranged in a 24-well dish. After the formation of the monolayer, a sterile pipette tip was used to create a scratch in the cell layer. Subsequently, the dish was positioned in an incubation chamber for 48 h to facilitate cellular migration and wound healing. At both 0 and 48 h, photographs were captured of the microscopic images of the injured area. Measuring the gap between the wound edges at various time intervals determined the rate of migration.

### 2.15 Cell death assays

Seed the cells onto a 6-well plate 24 h in advance. Following transfection with si-RNA and a 48-h incubation period, the cells were detached using trypsin and collected in 1.7-ml microtubes. Subsequently, they were washed with PBS and suspended in cold PBS containing 2 μm propidium iodide (PI). Flow cytometry analysis was conducted using a BD Accuri C6 instrument (BD Biosciences) and the FlowJo v10 software to identify and quantify the population of dead cells (PI-positive).

### 2.16 Real-time RT-PCR

A qRT-PCR and ChamQ SYBR qPCR mix kit (Q311-02; Vazyme) was used. Relative expression of target gene was determined by the 2^-ΔΔCt formula. This formula allows for the normalization of gene expression levels to an internal control and comparison between different experimental conditions. For qRT-PCR, the primers utilized were CSF1 Forward Primer (5′-TGGCGAGCAGGAGTATCAC-3′) and Reverse Primer (5′-AGGTCTCCATCTGACTGTCAAT-3′).

### 2.17 Statistical analysis

The data of experimental was analyzed using GraphPad Prism 5. Bioinformatics analysis, excluding descriptive analysis, were performed using R language (version 4.2.1). We adjusted p-values, including the false discovery rate, using *t*-test, two-way ANOVA, and the Benjamini–Hochberg method. p-value <0.05 was regarded statistically significant.

## 3 Results

### 3.1 Identification of the prognostic DEGs

The ten disulfidptosis-related genes [9] from the LUAD samples were regarded as DEGs, as shown in Supplementary Table 2. Univariate Cox regression for the ten disulfidptosis-related genes and selected four prognostic disulfidptosis-related genes based on TCGA cohort’ OS (Fig. 1A). We then employed a Venn diagram and heatmap to identify the four disulfidptosis-related prognostic DEGs (Fig. 1B and C). We computed the Pearson correlation coefficient of the four prognostic DEGs to evaluate the potential interactions between these genes using a cutoff value of 0.2 (Fig. 1D), which showed that NCKAP1 was positively related to the other three genes (GYS1, SLC7A11, and SLC3A2).

**Fig. 1:**
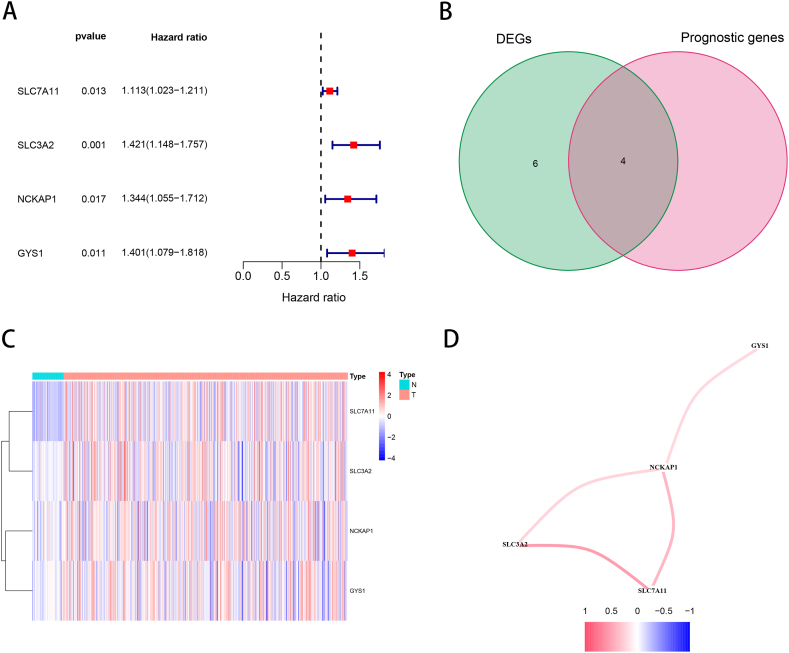
The expression and interaction of 4 overlapping disulfidptosis-related genes (A) The 4 prognostic disulfidptosis-related genes in LUAD, (B) venn chart, (C) expression of the 4 overlapping genes in LUAD, (D) correlation network between 4 intersectional genes, p < 0.05 was considered significant.

### 3.2 Construction of the prognostic model

**Fig. 2:**
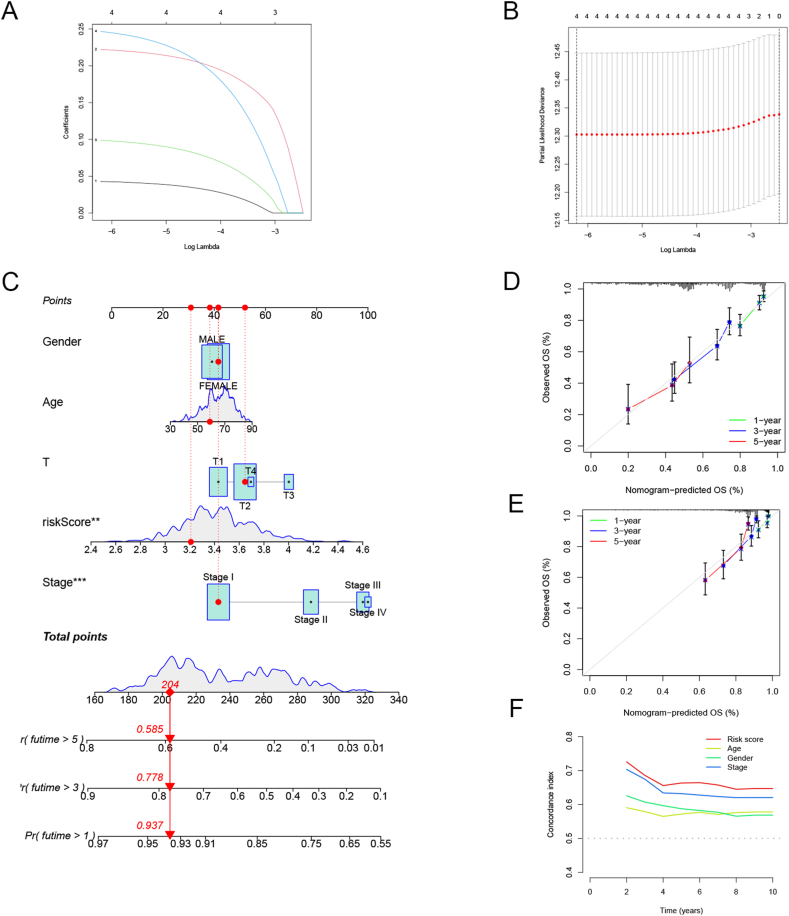
Establishment of the training group prognostic model. (A) Four overlapping genes' LASSO coefficients, (B) 10-fold cross-validation. (C) A nomogram of prognostic signature. (D, E) The calibration curves to validate the nomogram for predicting 1-, 3- and 5- years survival of the patients. (F) The concordance index (*C*-index), ranges from 0.5 to 1.0; the higher the C index, the better the ability to differentiate.

We developed a prognostic model for LUAD that relies on four genes associated with disulfidptosis via the LASSO algorithm. Risk score calculation method: risk score = (0.0428518 × exp (SLC7A11)) + (0.2221021 × exp (SLC3A2)) + (0.2466292 × exp (GYS1)) + (0.0987988 × exp (NCKAP1)). Two distinct groups: high-risk (N = 251) and low-risk (N = 252) were categorized based on risk score derived from the TCGA-train cohort. This division was based on the relative risk scores assigned to each patient, allowing for a clear differentiation between individuals with higher and lower risk profiles. Supplementary Table 3 depicts the selection of LASSO parameters. The LASSO regression algorithm identified four genes associated with OS based on the optimal λ value and the minimum partial likelihood of deviance (Fig. 2A and B). The nomogram, which incorporates stage, risk score, age, and sex and predicts the 1-, 3-, and 5-year OS of LUAD (Fig. 2C). Calibration curves (Fig. 2D and E) were consistent in observing and survival predicting in both the training and validation groups, indicating that the nomogram is reliable in predicting survival outcomes in LUAD patients at these time points. *C*-index curve showed that the prognostic model-related risk-scores had high discriminatory power and a brilliant degree of accuracy across a wide range of clinical information (Fig. 2F).

**Fig. 3:**
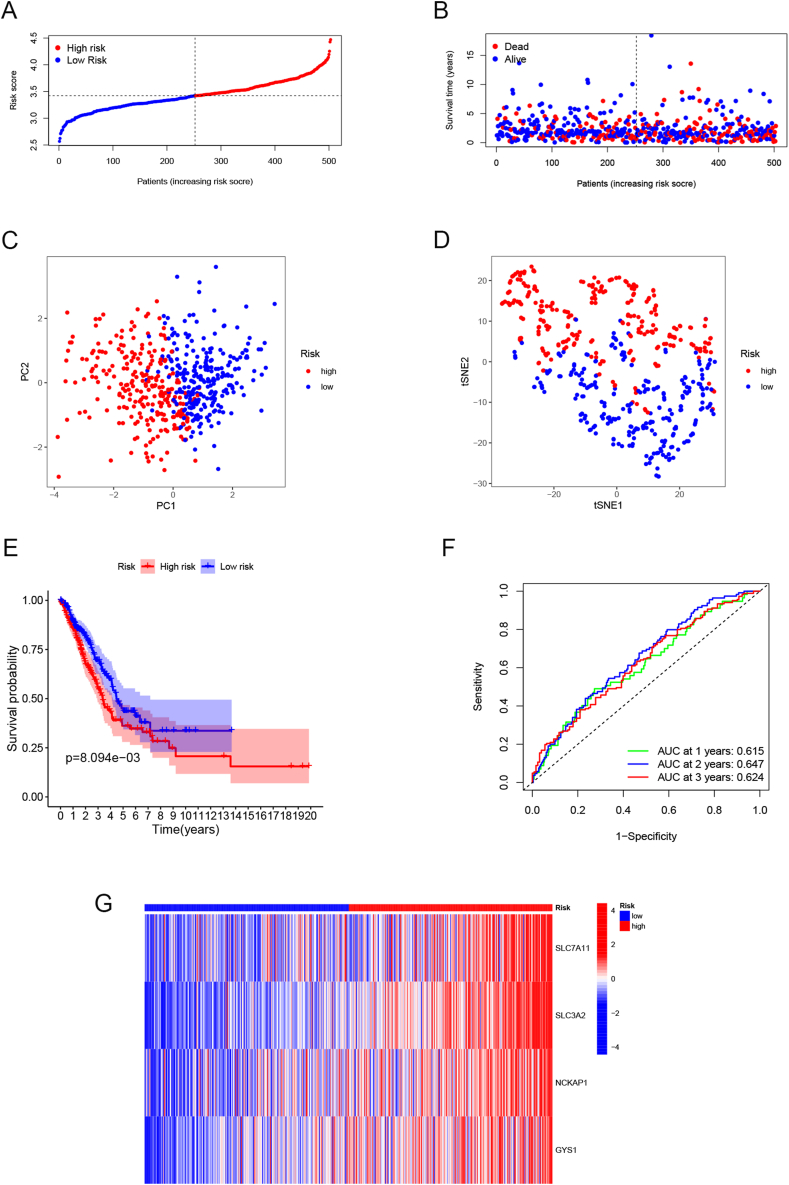
TCGA cohort prognostic signature construction. Distribution of risk score in TCGA-LUAD samples' (A) OS and (B) status. (C) PCA, (D) *t*-SNE analysis of the training group. (E) Kaplan-Meier survival and (F) ROC curve analysis of the four disulfidptosis-related genes signature. (G) Heatmap, expression profiles of the four disulfidptosis-related genes in high/low-risk groups.

Training cohort samples were stratified into subgrops with high and low risk (Fig. 3A), where the high-risk subgroup had a higher likelihood of experiencing mortality (Fig. 3B). Assess the effectiveness of the signature by conducting PCA, *t*-SNE, and ROC analyses. In Fig. 3C and D, both PCA and *t*-SNE revealed that the prognostic signature had a remarkable capability to successfully distinguish patients into high-/low-risk categories. Additionally, low-risk group displayed a better OS outcome in Kaplan-Meier curve (Fig. 3E, p < 0.001). In Fig. 3F, the area under the curve (AUC) was 0.617, 0.648, and 0.624 at 1, 2, and 3 years, respectively. The expression levels of the four differentially regulated genes in each sample between the two risk groups (Fig. 3G).

### 3.3 Validation in GEO cohort

**Fig. 4:**
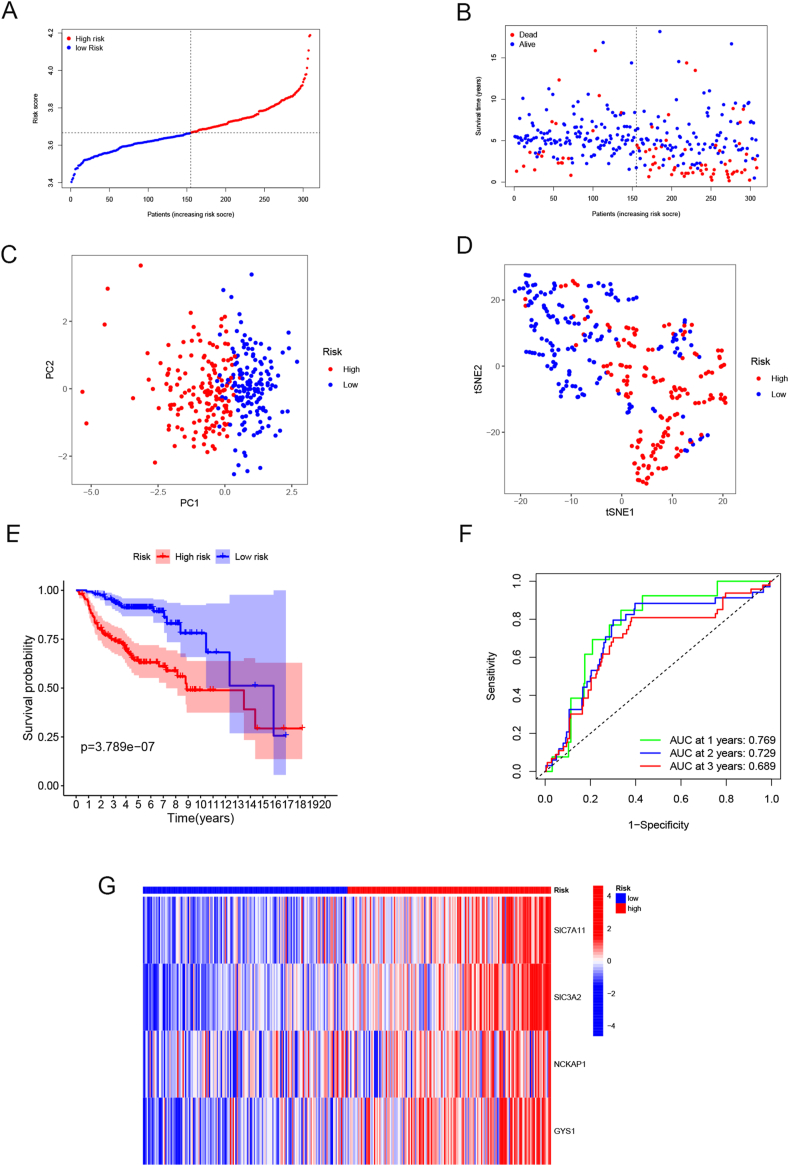
Prognostic signature's validation in GEO cohort. (A) Distribution of the risk-score in the validation group, (B) survival status and risk score of each patient. (C) PCA, (D) *t*-SNE analysis, (E) Kaplan–Meier survival curve. (F) ROC curve predicting efficiency of prognostic signature. (G) Expression profiles of the four-disulfidptosis-related genes in risk related groups in the GEO cohort.

The four-gene model established in the training group was applied to the merged cohorts from GSE30219 and GSE31210. In the merged GEO cohort, 309 samples were category into subgrops with high and low risk as well (Fig. 4A and B). Similarly, PCA and *t*-SNE analyses suggested distinct trends in the GEO cohort (Fig. 4C and D), consistent with training cohort. The Kaplan-Meier curve revealed that samples in low-risk group exhibited a better OS, same as the training cohort (Fig. 4E). The AUC reached 0.769, 0.729, and 0.689 at 1, 2, and 3 years, respectively (Fig. 4F). The heatmap depicted that the expression patterns of the four-gene signature in the high-/low-risk samples from the GEO validation group were consistent with those observed in the training group (Fig. 4G).

### 3.4 Independent prognostic analysis

**Fig. 5:**
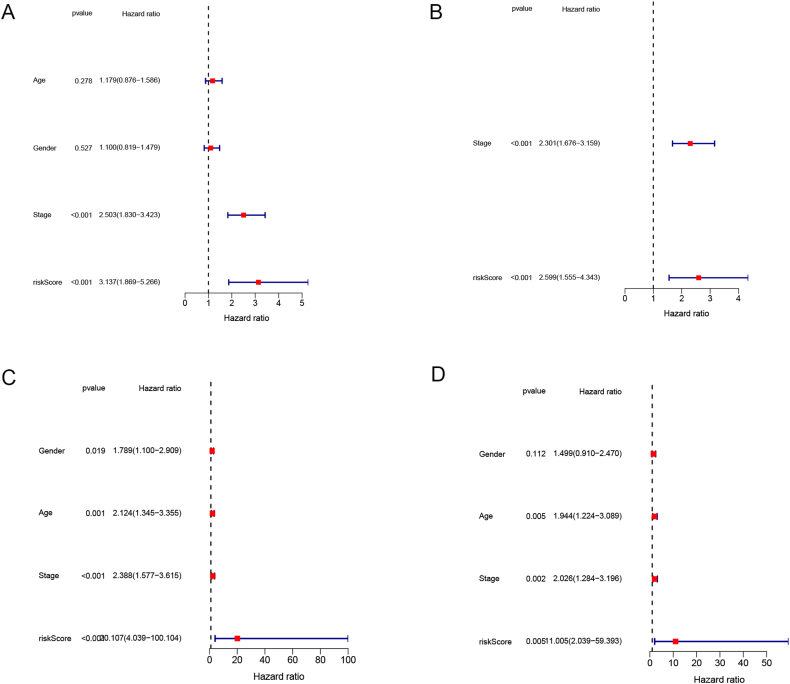
Independent prognostic analysis. TCGA cohort (A) univariate Cox regression, (B) multivariate Cox regression. (C) GEO cohort univariate Cox regression, (D) multivariate Cox regression.

Univariate Cox regression in training cohort identified stage, age, sex, and risk score to be clinically significant (Fig. 5A). However, the significant prognostic factors identified through multivariate analysis were the stage and risk score (Fig. 5B). The merged GEO cohort included individuals of different ages, genders, stages, and risk-scores. Both multivariate and univariate Cox regression analyses demonstrated the statistical significance of the risk score and stage (Fig. 5C and D).

### 3.5 Functional enrichment analysis

**Fig. 6:**
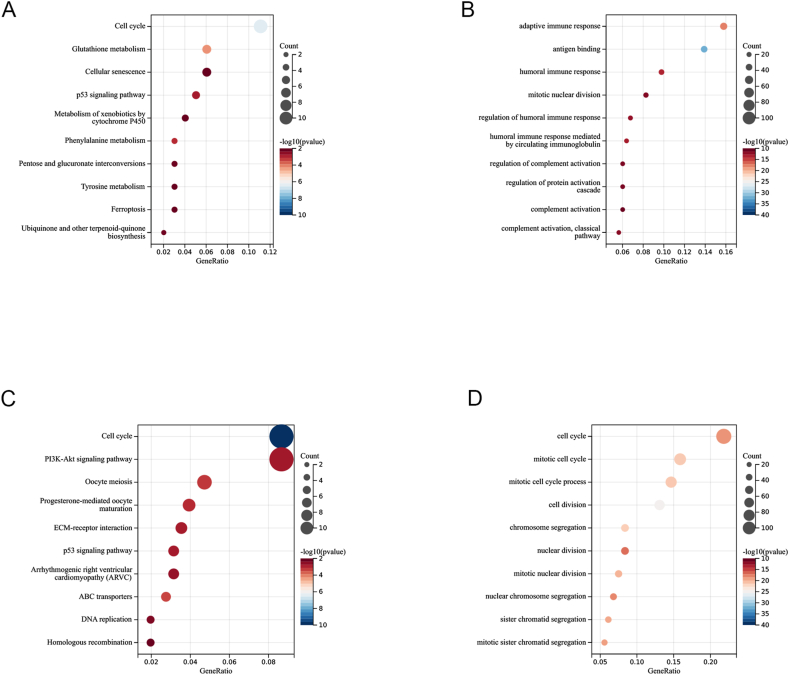
Analysis of functional enrichment. The risk score-associated DEGs' GO and KEGG pathways were analyzed in the training cohorts (A, B) and testing cohorts (C, D).

A total of 432 and 704 risk-related differentially expressed genes were revealed in training and validation cohort, respectively, using Wilcoxon test (Supplementary Tables 4 and 5). We found that these genes in the training group were primarily associated with the adaptive immune response, antigen binding, mitotic nuclear division, and other related processes (Fig. 6A). In addition to the functional pathways mentioned above, we observed that risk-related DEGs were enriched in nuclear and cell division processes in the validation group (Fig. 6C). P53 and the cell cycle were among the KEGG pathways enriched in both cohorts (Fig. 6B, D).

### 3.6 Immune analysis

**Fig. 7:**
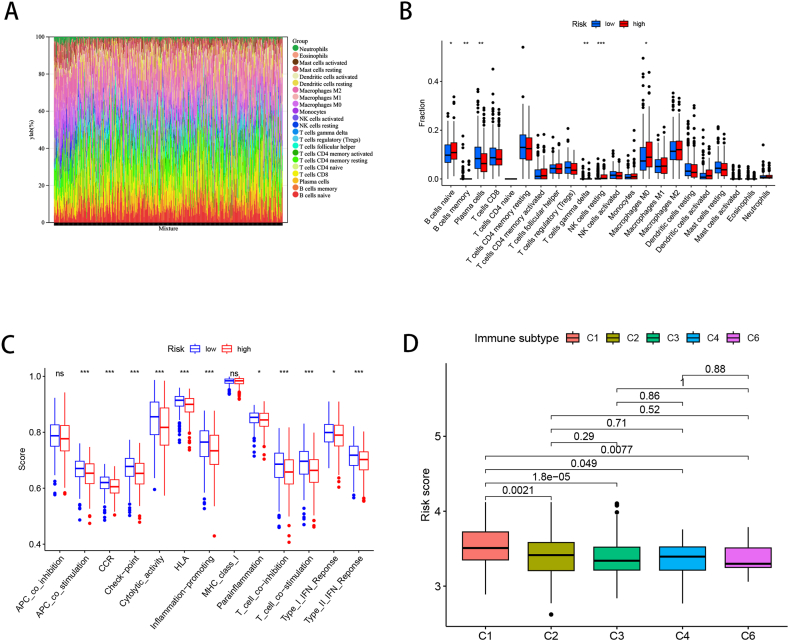
The immune infiltration and function analysis in training cohort. (A) Stacking diagram was generated to illustrate the immune infiltration of immune cells in TCGA-LUAD patients using the CIBERSORT algorithm. (B) Immune infiltration levels Box-plot. (C) The immune function analysis based on ssGSEA. (D) Correlation of risk scores between different immunophenotypes (p < 0.05).

CIBERSORT algorithm for analyzing differences in immune infiltration in two risk subgroups. There was an increase in macrophages and a smaller proportion of natural killer cells in morphologically diverse samples (Fig. 7A). Immune cell differential analysis showed that CD8^+^ T cells, CD4^+^ memory resting T cells, and plasma cells, were reduced in the high-risk group, whereas M0, M1, and M2 macrophages and naive B cells were reduced in the low-risk group (Fig. 7B). Immune function, type II interferon response, T-cell co-inhibition, cytolytic activity, and HLA levels were reduced in the high-risk group (Fig. 7C). Fig. 7D shows that only the C1 wound-healing immune subtype was associated with the others.

### 3.7 The signature predicts the efficacy of response to chemotherapy and immunotherapy

**Fig. 8:**
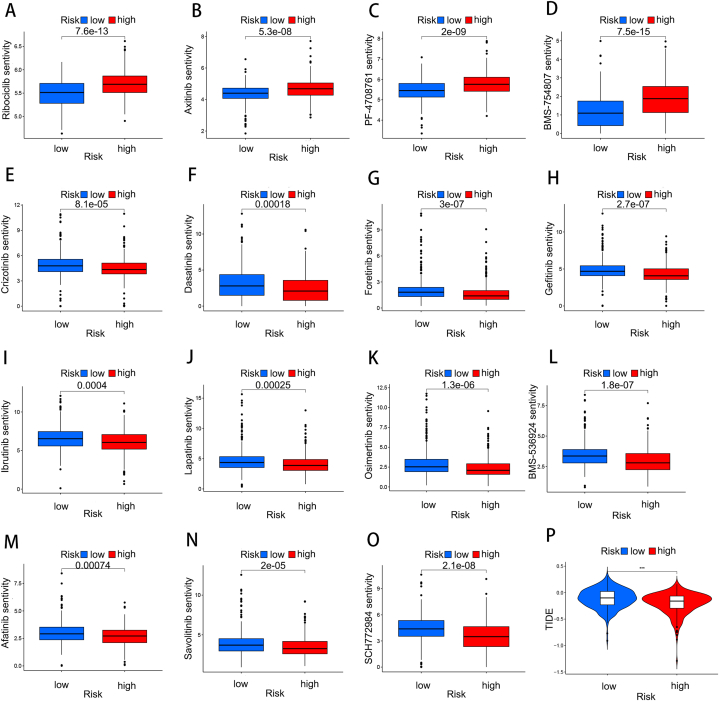
Signature chemotherapy and immunotherapy prediction. High-risk scores and lower IC50 for chemotherapeutics such as (A) Ribociclib, (B) Axitinib, (C) PF-4708671, (D) BMS-475807, whereas they were related to a higher IC50 for (E) Crizotinib, (F) Dasatinib, (G) Foretinib, (H) Gefitinib, (I) Ibrutinib, (J) Lapatinib, (K) Osimertinib, (L) BMS-536924, (M) Afatinib, (N) Savolitinib and (O) SCH772984 treatment. (P) TIDE score.

To evaluate whether our model was related to LUAD drug therapy, we conducted an analysis to compare drug effectiveness between two risk groups. A notable correlation between the low-risk group and higher IC50 values in response to chemotherapy drugs, such as ribociclib, axitinib, PF-4708671, and BMS-754807 (Fig. 8A–D). Conversely, the high-risk group demonstrated greater sensitivity to crizotinib, dasatinib, SCH772984, ibrutinib, BMS-536924, lapatinib, afatinib, gefitinib, foretinib, and savolitinib (Fig. 8E–O). We used TIDE scoring to assess immunotherapy tolerance among the different risk groups. The findings showed a noteworthy discrepancy in TIDE scores between groups with highand low risk, with the former exhibiting lower scores (Fig. 8P). These findings suggest that patients with low risk exhibit higher tolerance to ICB and more likelihood of immune evasion; therefore, high-risk patients should show a better effect to ICB treatment.

### 3.8 Validation of expression levels and function of signature genes

**Fig. 9:**
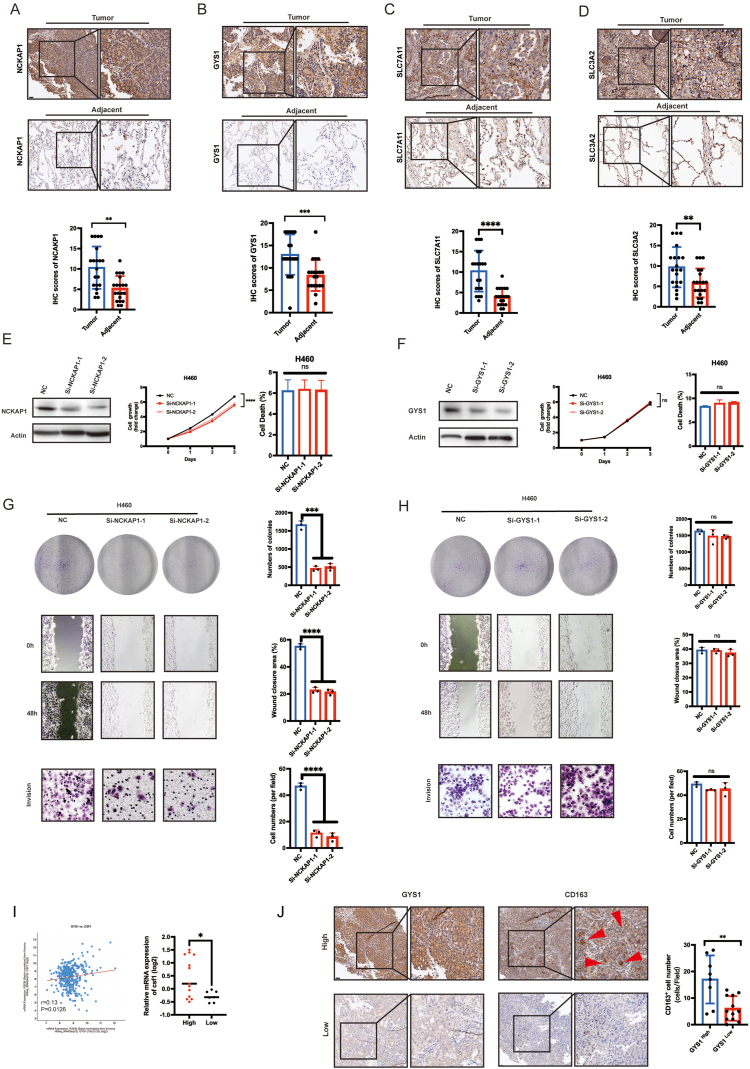
Evaluation of SLC7A11, SLC3A2, NCKAP1 and GYS1 expression and prognosis value in LUAD. (A–D) Immunohistochemical staining of NCKAP1, GYS1, SLC7A11, SLC3A2 in clinical LUAD samples between tumor and adjacent tissues (scale bars, 20 μm). (E) Immunoblot of NCKAP1 in lung cancer cells. CCK-8 assay results showed the NCKAP1 knockdown cell proliferation in H460 cell lines, quantification of cell death after NCKAP1 knockdown by flow cytometry. (F) Immunoblot analysis of GYS1 in lung cancer cells. CCK-8 assay about GYS1 knockdown cell proliferation in H460 cell lines, quantification of cell death after NCKAP1 knockdown by flow cytometry. (G) NCKAP1 inhibited cell growth, migration and invasion in H460. Cell colony formation; wound-healing assay: *Si*-NCKAP1-1 and *Si*-NCKAP1-2; transwell invasion showed a decrease of invaded knockdown cells. Scale bar = 20 μm. (H) GYS1 inhibited cell growth, migration and invasion in H460. Cell colony formation; wound-healing: *Si*-GYS1-1 and *Si*-GYS1-2; transwell invasion assay results showed a decrease of invaded knockdown cells. Scale bar = 20 μm. (I) Correlation between GYS1 expression level and infiltration level of CSF1, quantitative analysis of CSF1 using real time PCR in GYS1 high and low expression groups. (J) IHC staining of GYS1 high/low expression and CD163 infiltration in LUAD tissue, scale bar = 20 μm.

Firstly, we examined the disparities in the manifestation of four predictive signature genes in lung cancer and paraneoplastic through IHC, and found that these genes were higher expressed in LUAD tumor tissues than adjacent counterparts (Fig. 9A–D). The impact of SLC7A11 [23] and SLC3A2 [24] on the prognosis of lung cancer have been documented, it is still uncertain whether NCKAP1 and GYS1 influence the prognosis in lung cancer remains unclear. Therefore, the next step of our research was to investigate their effects on the prognosis of lung cancer cells.

The expression levels of NCKAP1 and GYS1 were examined in both lung cancer and normal alveolar epithelial cells (Supplementary Fig. 1). To investigate the functional roles of NCKAP1 and GYS1, we conducted experiments using the human lung cancer H460 cell line with knockdown of NCKAP1 and GYS1. Knocking down NCKAP1 led to a reduction in the rate of cell growth (Fig. 9E, Supplementary Fig. 2E) and a decline in the quantity and magnitude of colonies formed compared with the vector control group (Fig. 9G). Additionally, the knockdown of NCKAP1 led to reduced wound closure and invasiveness (Fig. 9G) compared to the vector control group. NCKAP1 may play some roles in regulating the proliferation, migration, and invasive capacity of LUAD cells. On the other hand, the knockdown of GYS1 did not show any significant effects on these cellular processes (Fig. 9F, H, Supplementary Fig. 2F). In the TME, immune cell infiltration is a crucial factor affecting tumors [25]. Therefore, we investigated the influence of GYS1 on immune infiltration. The correlation between GYS1 and CD8^+^ T cells Tregs and macrophages was analyzed using Timer 2.0 [15]. The results revealed that GYS1 was not correlated with CD8^+^ T cells (p > 0.05, R = 0.031), but was positively correlated with Tregs (p < 0.05, R = 0.031) and macrophage infiltration (p < 0.05, R = 0.333). We then utilized the cBioPortal database to analyze the assoiation between the macrophage chemokines CSF1 and CCL5 (Supplementary Fig. 2D). GYS1 expression was positively correlated to CSF1 (p = 0.0126, r = 0.13) and CCL5 (p < 0.001, r = 0.22). CSF1 infiltration in the high-/low-GYS1 expression groups was quantified using real-time PCR. The results demonstrated that CSF1 infiltration in the TME increased with increasing GYS1 expression (Fig. 9I). IHC with high or low GYS1 expression was performed on LUAD tissue chips, which indicated that the high-expression group showed significantly more macrophage (CD163) infiltration (Fig. 9J). The original Western blot (WB) images for Supplementary Fig. 1 will be included in the supplementary files for better visualization. The original Western blot images for Supplementary Fig 1 and Fig. 9 E, F have been included in the supplementary files for better visualization.

## 4 Discussion

Necrosis and apoptosis, two of the most frequently reported types of cell death [26]. Disulfidptosis, a recently identified form of RCD, has received little attention. Hence, the identification of pathways associated with disulfidptosis has crucial implications for understanding the mechanism of this form of cell death, discovering cancer drugs, and gaining deeper insights into related ailments. LUAD is a highly malignant cancer of the respiratory system. Despite substantial research efforts, efficient biomarkers for accurate diagnosis and prognosis remain scarce, thereby burdening the public healthcare sector.

Early diagnosis and of prognostic prediction are crucial for the management of LUAD. Early diagnosis is vital for better treatment outcomes and improved prognosis. On the other hand, prognostic models have a crucial function in predicting the disease progression and patient outcomes, aiding in personalized treatment decisions and follow-up strategies. Herein, we identified the disulfidptosis-related DEGs. Subsequently, using univariate regression and LASSO Cox regression, four disulfidptosis-related genes were incorporated into a novel prognostic risk signature. The signature is composed of SLC7A11, SLC3A2, GYS1, and NCKAP1, all of which are differentially expressed genes associated with disulfidptosis that exhibit a robust correlation with other regulators of the RCD process. The prognostic signature is useful for predicting patient prognosis, guiding treatment decisions, understanding tumor mechanisms, enabling personalized medicine, and facilitating drug development and clinical trial design. They provide accurate prognostic assessments, aiding clinicians in tailoring individualized treatment plans and improving patient outcomes. Additionally, these models shed light on the molecular mechanisms and biological processes underlying tumor development, leading to the discovery of novel therapeutic targets and prognostic markers. The prognostic model constructed through the above process is the core of this study, and all other analyses conducted in this research revolve around this prognostic model. By predicting the prognosis of LUAD patients, targeted personalized treatment plans can be developed, leading to extended survival time and improved quality of life. The analysis of drug sensitivity and immune infiltration, offers potential strategies to enhance treatment outcomes in LUAD patients. Based on this model, we established a nomogram that incorporated factors, such as risk score, sex, T stage, and age from the signature (stages N and M were excluded because of uncertain values). Risk scores, independent risk factor for predicting prognosis in LUAD patients. ROC curves and survival analysis revealed that this disulfidptosis-related signature accurately differentiated between high-/low-risk populations and reliably predicted prognosis in patients with LUAD. By measuring the expression levels of the four core genes associated with this prognostic signature in LUAD patients, it is possible to make certain predictions regarding their prognosis from the perspective of disulfidptosis.

Immune infiltration analysis and potential drug sensitivity validated the expression levels of model genes to better assess the prognosis of LUAD. Functional enrichment analysis revealed that DEGs linked to risk scores were enriched in epithelial cell proliferation and nuclear division. GO analysis showed that the risk-related DEGs associated with risk scores were mainly enriched in the humoral and adaptive immune responses. These results indicated that disulfidptosis-related genes may control the advancement and progression of LUAD by regulating these critical pathways. Furthermore, these risk-related DEGs were enriched in the cell cycle, p53 signaling, pathways, all of which are critical for the development of the disulfidptosis-related regulator SLC7A11 [[27], [28], [29]]. As a tumor suppressor, p53 exerts tumor suppressor effects by inhibiting the cell cycle and inducing apoptosis [30]. SLC7A11 is a key gene for disulfidptosis and ferroptosis, and previous studies have indicated that p53 can promote ferroptosis by suppressing the expression of SLC7A11 [31]. Moreover, disulfidptosis is a unique form of RCD that occurs during a lack of glucose and is characterized by elevated levels of SLC7A11 [9]; hence, there may be a relevant mechanism for the promotion or inhibition of disulfidptosis by p53. The Nck protein family, known as regulators of the actin cytoskeleton, are considered adapter proteins. The bonding of intracellular disulfides to actin cytoskeleton proteins leads to the emergence of a novel cell death model known as disulfidptosis [32]. It has also been reported that key genes of disulfidptosis, such as SLC7A11 [31], NCKAP1 [33] and SLC3A2 [24], may be involved in the regulation of cell cycle. It is possible that there is a mechanism that affects the cell cycle through disulfidptosis-related genes, either alone or in concert. There may be a mechanism by which disulfidptosis is promoted or inhibited by p53 and cell cycle; however, this needs to be demonstrated by more in-depth studies.

The low-risk group exhibited a higher TIDE score in our investigation. Nevertheless, additional research is necessary to ascertain the impact of immunotherapy on individuals diagnosed with LUAD, specifically in relation to prognostic signatures associated with disulfidptosis. Renal clear-cell carcinoma shows a positive correlation with immune cells in relation to NCKAP1. Moreover, NCKAP1 expression is significantly correlated with various immune checkpoint markers including HAVCR2, CTLA4, TIGIT, CD274, LAG3, and PDCD1 [34]. Upregulating expression of SLC3A2 in immune cells may augment the efficacy of tumor immunotherapy [35]. The OncoPredict drug sensitivity study revealed that the high-risk group exhibited greater sensitivity to anticancer drugs. However, ribociclib, axitinib, PF-4708671, and BMS-754807 showed opposite trends. Axitinib, one of the most potent and latest anti-angiogenic tyrosine kinase inhibitors currently under evaluation for the treatment of NSCLC, is a highly selective inhibitor of VEGFR-1, -2, and -3 [36]. Inhibition by PF-4708671 in three NSCLC cell lines decreased the phosphorylation of p70S6K and its downstream S6 [37], which plays a keying action in phosphorylation and cell proliferation, and affects the cell cycle and invasion of NSCLC [38]. BMS-754807 inhibits kinases belonging to the insulin-like growth factor 1 receptors/insulin receptors [39]. Treatment with BMS-754807 alone enhanced apoptosis in human lung cancer cells, likely via IGF-IR/IR signaling. Given these properties, BMS-754807 may be a latent therapeutic candidate for lung cancer treatment, especially in patients with high IGF-IR expression levels in lung tumors [40]. Our study found that the above-mentioned drugs exhibited higher sensitivity in the low-risk group, which warrants further investigation and research into drug combinations and detection of drug sensitivity in the in vitro environment.

Disulfidptosis is mainly found in cells during glucose starvation with high expression of SLC7A11 because of the inability of the NADPH supply to meet the requirements of the process of cystine reduction to cysteine, which causes rapid cell death by disulfide stress. Transporting cystine, SLC7A11 acts as a vital cystine/glutamate reverse transporter, contributing significantly to the production of glutathione and the protection against oxidative stress. Overexpression of it is common in human cancers [41]. SLC7A11 and SLC3A2 were the two most highly upregulated genes in disulfidptosis and played a crucial role in our model. Experimental verification of previous research on SLC7A11 [42] and SLC3A2 [24] was conducted in NSCLC to verify our validation. SLC7A11 has been reported to promotes the progression of NSCLC through metabolic reprogramming, and SLC7A11 be associated with lethality in KRAS-mutant LUAD. In lung cancer cells, tumorigenesis was triggered by SLC3A2 through the MEK/ERK signaling pathway. However, the expression and role of NCKAP1 and GYS1 in lung cancer remain unclear. NCKAP1 alone boosts the invasion and metastasis of NSCLC [43]. Our experimental results confirmed this point of view and revealed that NCKAP1 also affects tumor cell migration, proliferation and clone formation in LUAD.

GYS1 is a pivotal rate-limiting enzyme that operates during the final stage of glycogen synthesis [44,45] showed that GYS1 is upregulated in lung cancer cell lines following radiotherapy. These findings suggested that GYS1 plays a crucial role in lung cancer metabolism. Our observations did not reveal any effect of GYS1 on lung cancer cell proliferation. However, the results of our study suggest that it is associated with macrophage and Treg infiltration. Recent studies have demonstrated that the role of cancer cells in TME and their interaction were vital in the development of drug resistance and tumor malignancy [46]. Chemokines are critical for regulating tumor activity and tolerance status, abundance of TILs cellular differentiation in pro- and anti-tumor immunoreactivity [47,48]. Further analysis implied that high GYS1 expression may be associated with a higher number of CD163-positive cells. CD163 is a protein unique to macrophages, and its elevated expression in macrophages is a hallmark of tissue response to inflammation [49]. Macrophage CSF1 is the principal growth factor that is essential for regulating the differentiation, proliferation, survival, and rejuvenation of macrophages [50]. The positive correlation between CSF1 and GYS1 suggests that GYS1 might promote LUAD progression by recruiting macrophages. Moreover, CCL5 can polarize macrophages into M2 macrophages by recruiting them, and this has been shown in patients with LUAD [51]. Tregs present in the tumor showed a notable rise, whereas they were not detectable in the surrounding healthy lung tissues. Furthermore, there was a discovered correlation between the quantities of Tregs and the manifestation of CCL5 and its receptor CCR5 [52]. An elevated level of CCL5 expression in LUAD was linked to a rise in Tregs and unfavorable survival rates [53]. However, it remains unclear whether these disulfidptosis-related genes affect the prognosis of patients with LUAD through their involvement in disulfidptosis or immune cell infiltration. In summary, knocking down NCKAP1 and GYS1 did not show any difference in cell death as measured by flow cytometry. These results indicate that under normal conditions, NCKAP1 and GYS1 do not affect cellular death. Disulfidptosis, a specific form of cell death, only occurs under certain conditions: glucose starvation and high expression of SLC7A11. Apart from that, under what conditions can lead to disulfidptosis? This poses a new question regarding disulfidptosis. Our study did not investigate the role of disulfidptosis in LUAD under glucose starvation conditions, for the occurrence of RCD may be influenced by various factors, but under normal conditions, disulfidptosis does not occur. Under glucose starvation conditions alone, disulfidptosis does not occur [9]. It is known that prolonged glucose starvation conditions can induce cell apoptosis even without high expression of SLC7A11, suggesting that NCKAP1 and GYS1's regulation may not necessarily involve disulfidptosis in the absence of high SLC7A11 expression [9,54,55]. It remains unknown whether NCKAP1 and GYS1 affect prognosis in LUAD through disulfidptosis. Additionally, it is important to note that glucose starvation alone is insufficient to trigger disulfidptosis. The influence of NCKAP1 and GYS1 on disulfidptosis induction during glucose starvation will be a key focus of our future research. In the absence of conditions inducing disulfidptosis, the impact of NCKAP1 on the prognosis of LUAD may be attributed to its promotion of proliferation, invasion, and migration, while the effect of GYS1 on LUAD prognosis may be related to its influence on immune infiltration in TME. Investigating the role of disulfidptosis genes in immune cells is also one of the future directions.

For the first time, we performed a comprehensive examination of LUAD data obtained from an openly accessible online database. Our analysis focused on disulfidptosis-related genes and their potential as prognostic indicators for LUAD. However, there were certain constraints in this research. First, the prognostic model constructed using the TCGA data had an AUC of less than 0.65 at 1 year for the training cohort. This was supplemented with the remaining analyses such as the c-index and calibration curves and the addition of external experiments to confirm the screened prognostic genes and their prognostic value. Second, the mechanism of disulfidptosis-related genes in LUAD prognosis needs to be further investigated. Third, the TCGA data included in this study had disparate numbers of tumor versus normal tissue samples; however, this was due to the limitations of the original TCGA samples. In an attempt to tackle this problem, this study used a validation set in the hope of remedying the problem of uneven distribution of the TCGA data.

This prognosis signature related genes identified through the construction of the prognostic model in this study have potential prognostic values. The selection was based on a bioinformatics approach that incorporated prognostic information and combined differential expression and Cox analysis. The prognostic value was confirmed through model construction, risk scoring, and subsequent analysis. Experimental verification demonstrated high expression of the model genes in tumor samples, and the combination of our results with published literature further supported their prognostic value. However, the mechanisms of action remain unclear and require comprehensive analysis in future studies.

## 5 Conclusion

Analyzing data from an online open database, this study is the initial attempt to assess and summarize the prognostic significance of genes related to disulfidptosis for LUAD using a systematic approach. Additionally, the protein expression of SLC7A11, SLC3A2, NCKAP1, and GYS1 was assessed highly expressed in clinical LUAD tissues. Specificly, NCKAP1 may affect prognosis by influencing tumor proliferation, invasion, and migration, while GYS1 may affect prognosis by influencing the immunosuppressive microenvironment. In summary, the prognostic model developed in this study can predict survival and prognosis in LUAD and is a signature of relevant ICB drug sensitivity and tumor immunological features.

## Ethics declaration

The studies involving human participants were reviewed and approved by Shanghai Outdo Biotech Company ethical committee (HLugA060PG02).

## Data availability statement

All data collected and analyzed in this study could be downloaded from public databases including TCGA (https://portal.gdc.cancer.gov/), GEO (https://www.ncbi.nlm.nih.gov/geo/), TIMER2.0 (http://timer.comp-genomics.org/), cBioportal (https://www.cbioportal.org/), TIDE (http://tide.dfci.harvard.edu/).

## Funding

This study was supported by grants from the 10.13039/501100001809National Natural Science Foundation of China, China (No. 82103467), 10.13039/501100015401Key research and development projects of Shaanxi Province, China (No.2022SF-026), Basic medicine and clinical integration innovation project of Xi'an Jiaotong University (No. YXJLRH2022033).

## CRediT authorship contribution statement

**Yanpeng Zhang:** Conceptualization. **Jingyang Sun:** Writing – original draft, Writing – review & editing. **Meng Li:** Conceptualization. **Liren Hou:** Validation. **Zhiyu Wang:** Validation. **Huanhuan Dong:** Formal analysis. **Wenjun Xu:** Software, Validation. **Rongxuan Jiang:** Data curation. **Yuhan Geng:** Writing – original draft. **Chungen Guan:** Investigation. **Zijiang Zhu:** Writing – original draft. **Hongyi Wang:** Data curation. **Qiuyu Gong:** Data curation. **Guangjian Zhang:** Conceptualization, Project administration.

## Declaration of competing interest

The authors declare that they have no known competing financial interests or personal relationships that could have appeared to influence the work reported in this paper.

## Figures and Tables

**figs1:**
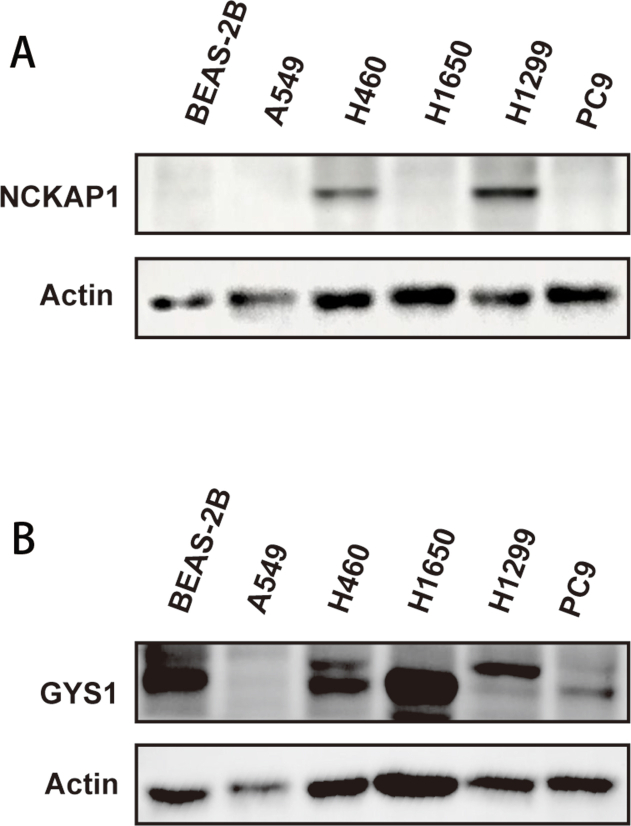


**figs2:**