# Supplementary figures and images for: Identification and validation of a disulfidptosis-related genes prognostic signature in lung adenocarcinoma
Source: Heliyon. 2023 Dec 19;10(1):e23502. doi: 10.1016/j.heliyon.2023.e23502 (PMC10784160; doi:10.1016/j.heliyon.2023.e23502)

Fig western blot

Fig 9 E, F:


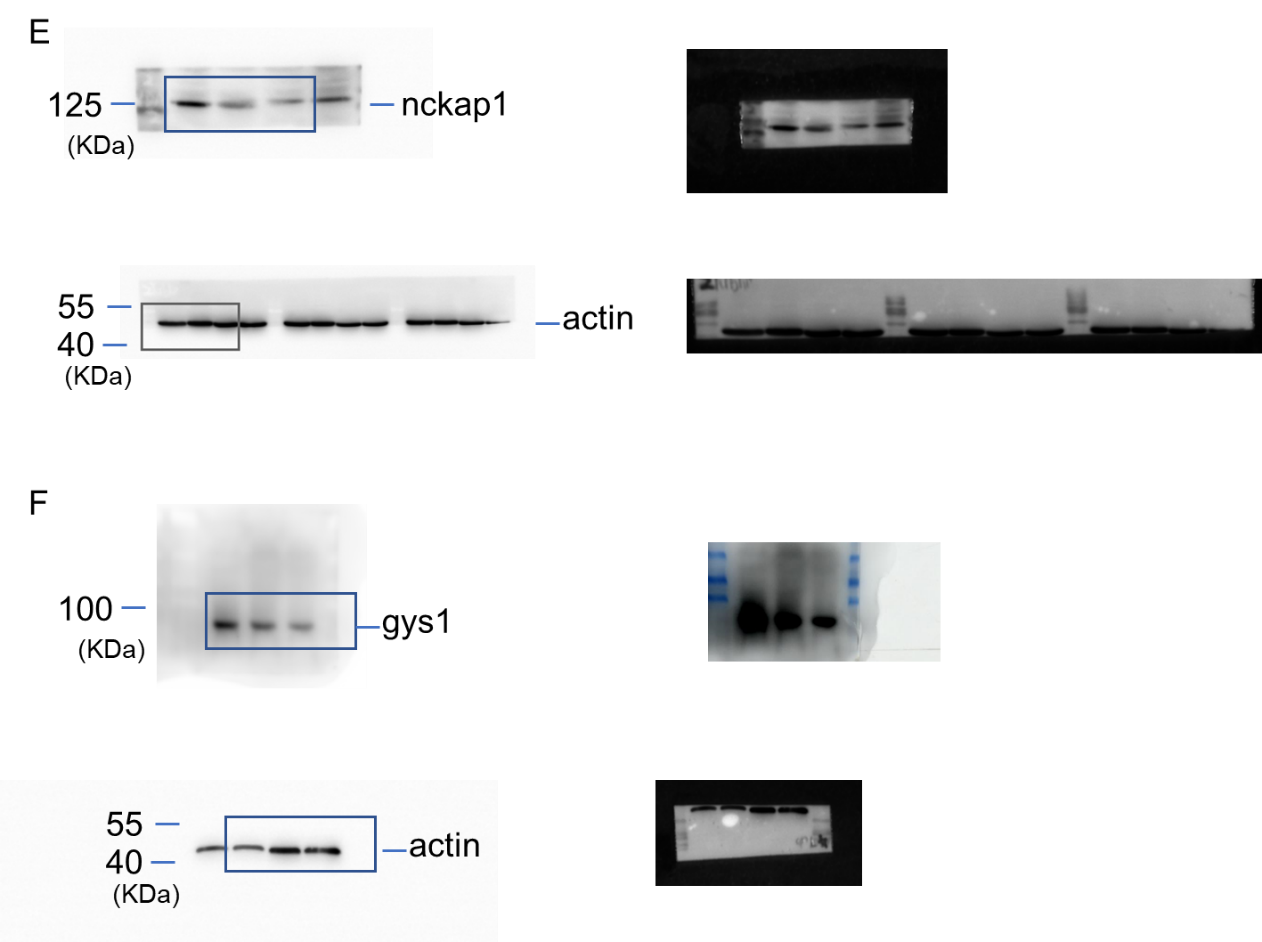


Supplementary Fig 1:


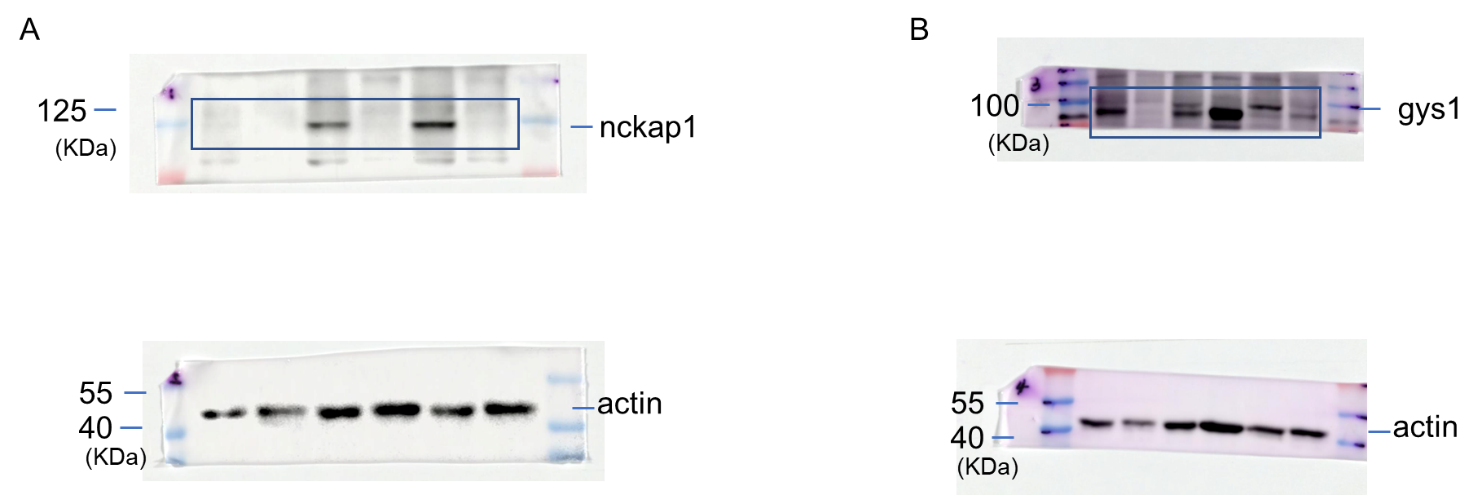

Supplement: Multimedia component 1 [file mmc1.docx]
